# Adverse Childhood Experiences and Depression among Homeless Young Adults: A Social Determinants of Health Perspective

**DOI:** 10.3390/ijerph21010081

**Published:** 2024-01-11

**Authors:** Shiyou Wu, Lac Ta, Jaime Vieira, Kendall Schwartz, Joshua Perez, Justin Zeien, Danyi Li, Jennifer Hartmark-Hill

**Affiliations:** 1 School of Social Work, Arizona State University, Phoenix, AZ 85004, USA; 2 College of Medicine–Phoenix, University of Arizona, Phoenix, AZ 85004, USA; talm@arizona.edu (L.T.); jvieira@arizona.edu (J.V.); kendallschwartz@arizona.edu (K.S.); jperez59@arizona.edu (J.P.); jhartmarkhill@arizona.edu (J.H.-H.); 3 Walter Reed National Military Medical Center, Bethesda, MD 20814, USA; justin.l.zeien.mil@health.mil; 4 Keck School of Medicine Preventive Medicine, University of Southern California, Los Angeles, CA 90032, USA; danyili@usc.edu

**Keywords:** adverse childhood experiences (ACEs), depression, social determinants of health, homeless, young adults, PHQ-4

## Abstract

Homelessness is a pervasive issue in the United States that presents significant challenges to public health. Homeless young adults (HYAs) are at particular risk for increased incidence and severity of depression. Using primary survey data (*n* = 205) collected in the Phoenix Metropolitan Area, Arizona, from June to August 2022, this study aims to examine the relationship between adverse childhood experiences (ACEs) and depression among HYAs. We adopted the ACEs 10-item scale to measure childhood traumatic experiences, whereas depression was measured by using a PHQ-4 depression scale and diagnosed depression. Regression models were conducted to test the relationships between ACEs and depression outcomes while controlling for the covariates at the individual, interpersonal, and socioeconomic/living environment levels. The average PHQ-4 score was 5.01 (SD = 3.59), and 59.69% of HYAs reported being diagnosed previously with depression. The mean ACEs score was 5.22 out of 10. Other things being equal, for every one unit increase in ACEs scores, the odds of being diagnosed with depression increased by 11.5%, yet it was not statistically significant, while the PHQ-4 score increased by 0.445 (*p* < 0.001). Overall, HYAs were disproportionately affected by depression. This study elucidates the complex relationship between ACEs and depression among HYAs.

## 1. Introduction

Homelessness is a pervasive, expanding issue in the United States that presents significant challenges to public health and social welfare [1]. People experiencing homelessness are more likely to be victims of violent crime and struggle with substance abuse disorders and untreated health issues, which can limit their access to social services [2]. Additionally, people experiencing homelessness may have fewer educational and job opportunities and may feel socially isolated [3]. The combination of these challenges can make it difficult for homeless individuals to break out of the cycle of homelessness and achieve stability and improved well-being. This study aims to investigate the risk and protective factors of depression among homeless population, and specifically focus on the associations between traumatic events experienced before the age of 18 (i.e., adverse childhood experiences [ACEs]) and depression among homeless young adults.

In 2022, the Annual Homeless Assessment Report to Congress from the Department of Housing and Urban Development demonstrated that an alarming number of people in the United States—approximately 582,000, or 18 out of every 10,000—experienced homelessness of at least one night’s duration [4]. According to the same report, Arizona ranks fifth in the nation for homelessness per capita and is currently experiencing the fifth fastest growing rate of homelessness relative to all other states. In addition, homelessness in Arizona youth populations has increased by nearly 45% between 2020 and 2022 [4]. Overall, these statistics necessitate further investigation into the root causes and contributing factors for homelessness in young adults.

Young adulthood is a critical period of physical, social, emotional, mental, and cognitive development [5,6,7]. Young adults make significant choices about their health behaviors, attitudes, and habits, which can have lasting effects over their lifetime [8]. Trauma, whether physical, mental, or psychosocial, that occurs during this timeframe can lead to changes in brain chemistry and neural connections, with consequential implications for future chronic illnesses. Furthermore, it is suggested that homelessness can increase all-cause mortality and decrease life expectancies by up to 30 years (average 42–52 years old) compared to those not experiencing homelessness [9,10,11].

The causes of homelessness among young adults are complex and varied [12,13,14,15]. Homeless young adults’ behaviors are influenced by factors across multiple domains (e.g., individual, interpersonal, and institutional levels), as understood from a social determinants of health (SDoH) framework [16,17]. This framework suggests that good health is produced by the complex interrelationships of genetic make-up, age, gender, behavioral factors, social determinants, and other environmental factors. An increasing number of studies have demonstrated a significant impact of social influences on an individual’s health status. Among other factors, SDoH include quality of housing, suitability of work, and access to healthcare and social welfare services [17,18,19]. These resource gaps can contribute to poor health [20].

### 1.1. Homelessness and Depression

Depression is a complex pathology that is associated with a spectrum of difficulties that interfere with daily life and activities [21]. It is important to note that current prevalence and incidence rates of depression may not accurately reflect true depression rates seen in young people [22]. Several challenges exist that inhibit individuals from finding help, including limited supply of mental health providers, limited access to care, and prohibitive costs of care [23]. Furthermore, it is likely that the recent COVID-19 pandemic has exacerbated this problem. Young adults lost many protective factors against depression including socialization, working opportunities, in-person activities, and environmental protective factors such as sunlight, exercise, and diet [24]. Additionally, they may have been exposed to increased risk factors such as toxic home environments and limited healthcare access. The totality of these complications, among others, makes data that accurately reflects the contemporary landscape of mental health near impossible to come by.

Previous research has suggested an increased prevalence of depressed mood in Hispanic and Black young adults when compared to White and Asian young adults [25]. This may be attributed to poorer educational prospects and problematic relationships [25]. Furthermore, minoritized populations may be less likely to utilize mental health services, worsening outcomes for these populations [26]. Females are also at increased risk for depression, especially in adolescence and young adulthood [27]. It is noteworthy that this discrepancy begins around the age of puberty.

Community-level stressors also play a significant role in depression. Factors with measured associations include poverty or violent neighborhoods, homelessness, and refugees/displacement [28]. Further, access to proper medical and mental healthcare may be limited, and resources may be instead spent on basic necessities of living [29]. While there is a genetic component to depression, its development and severity is heavily influenced by environmental factors, including demographics, that predispose people to constant stressors, significantly altering depression’s prevalence and severity [30]. This concept is of particular relevance when observing populations who face some level of social marginalization. 

The aforementioned demographic factors set the context for why homeless young adults are at particular risk for increased incidence and severity of depression. Homeless young adults are disproportionately made up of ethnic minorities, such as Blacks and Hispanics, and tend to come from impoverished backgrounds and face more stressors during development [31]. Add in the societal stigma tied to housing status within the United States, and the positionality of young adults experiencing homelessness becomes all the more complex. One way to bring clarity to the complexity associated with the homeless young adult demographic is through the use of adverse childhood experiences (ACEs) scores, which can help quantify the impact of the aforementioned stressors.

### 1.2. ACEs and Depression among Young Adults

ACEs are potentially traumatic events experienced between the ages of 0 and 17 that are demonstrated to negatively impact future health outcomes [32,33]. These experiences are divided into two categories: abuse and household dysfunction [33]. The research literature demonstrates a strong dose–response relationship between higher ACEs scores and increased risky health behaviors (e.g., smoking, drug use, physical inactivity, and risky sexual behavior), adverse health outcomes (e.g., liver disease, chronic lung disease, kidney disease, and arthritis), and psychosocial/behavioral measures (e.g., interpersonal violence, suicidal ideation, depressed mood, and behavior problems) [33,34,35,36]. Of note, ACEs scores higher than four are considered high risk, whereas ACEs scores of 1–3 are considered intermediate risk unless associated with other health conditions which raise the level of concern to high risk [37]. Similar to other SDoH, ACEs scores are also experienced in higher frequencies by individuals who identify as female, non-white, LGBTQIA+, of lower education level, and of lower socioeconomic status [34,38]. When further evaluating age discrepancies, one study found all correlates of ACEs scores to be higher in younger populations (ages 18–34), except for depression which was highest in the oldest population [39]. Studies that further divide this age group found those ages 25–34 had the highest scores [36,38]. In young adults, ACEs scores are correlated with mental health disorders including an increased risk of suicidal ideation [40] and depression [40,41,42]. 

Individuals experiencing homelessness struggle with a multitude of the aforementioned challenges; however, evaluating the past experiences of these individuals may provide further insight into the current morbidities they face. Consistent with other measures of health, ACEs scores are unsurprisingly elevated in individuals experiencing homelessness [43,44,45]. Additionally, although not based on the specific ACEs questionnaire developed by Felitti et al. [33], Herman et al. found that individuals experiencing homelessness had much higher rates of experiencing the ACEs of lack of parental care, physical abuse, and lack of parental care in tandem with either physical or sexual abuse compared to their housed counterparts [46]. While prevalence varies by study, some of the most common ACEs experienced by those that are experiencing homelessness include physical abuse, physical neglect, general household dysfunction, and emotional abuse [44,47]. Prevalence of ACEs scores amongst the homeless population is estimated to be 89.8% of individuals experiencing at least one ACE, and 53.9% experiencing four or more ACEs with significant associations with depression, suicidality, substance misuse, and victimization [45]. Sexual abuse is also prominent, with one study reporting 45% of women experiencing this form of abuse [44] and another study citing 33% of all participants experiencing sexual abuse [47]. Liu et al. found an average ACEs score of 4.1 for a homeless population in Canada, which is much higher than the aforementioned average, although the former was measured in the U.S. population [47]. Comparatively, in a study of indigenous individuals experiencing homelessness, the average ACEs score was 6.06 with 82% of individuals experiencing four or more ACEs [48]. Interestingly, this study found no significant differences between ACEs scores and health outcomes with the exception of an association between higher ACEs scores and mental illness [48].

Similar to the general population, ACEs are also predictive of poor mental health in those experiencing homelessness [44,45,47,48]. Roos et al. found a significant association between Axis I and Axis II Disorders (categorized in the DSM-IV as Mental Health/Substance Abuse and Personality Disorders/Mental Retardation, respectively, and have since been removed in the DSM-V) and ACEs scores with these disorders having a mediator effect on the risk of lifetime homelessness, although ACEs alone were also predictive [44]. ACEs scores are associated with a broad range of psychiatric disorders in the homeless population such as PTSD, mood disorder with psychotic features, depression, suicidality, and substance dependence [47,49,50]. However, depression more specifically has demonstrated mixed results with some studies reporting significant associations with total ACEs score [49,50], and others finding no relationship [47]. Additionally, no study to our knowledge specifically examines depression in regard to ACEs scores in a young homeless population. To address this literature gap, this study will use first-hand data to explore the mechanisms underlying the relationship between ACEs and depression among homeless young adults. 

## 2. Materials and Methods

### 2.1. Survey and Sample

The current study uses survey data from the Social Determinants of Health among Homeless Young Adults (SDoH-HYA) pilot project in the Phoenix, Arizona metropolitan area. The metropolitan Phoenix area is defined as the downtown area of Phoenix and its surrounding cities, such as Chandler and Mesa. Survey sites consist of areas where community outreach partners provide services, including Street Medicine Phoenix, Salvation Army Chandler, Grace Lutheran Church (Phoenix), Phoenix Rescue Mission, and Solutions of Sobriety. In addition, we collected data at some Phoenix Heat Relief Network cooling center sites (e.g., Tumbleweed park, downtown Phoenix, and Chandler library), the Maricopa County Human Services Campus, and the surrounding encampments. Survey administrators included graduate students from Arizona State University (ASU) and medical students from the University of Arizona College of Medicine, Phoenix.

Participants were recruited at various aforementioned survey sites on variable days of the week from June to August 2022. For inclusion in the survey, we screened participants to find individuals who identified as homeless (i.e., a person who does not have a fixed, regular, and adequate nighttime residence), either sheltered or unsheltered, and were between the ages of 18 and 34. This study was reviewed and approved by the Arizona State University Institutional Review Board. Potential adult participants were identified and recruited at survey sites on varying days of the week, typically during the morning to early afternoon. 

A total of 205 participants completed the survey, utilizing both paper and online surveys. The SDoH-HYA survey was developed by researchers from ASU as a comprehensive tool to collect data regarding the social determinants of health based on the theoretical framework of the wider determinants of health model [17,51]. The questionnaire items were reviewed and categorized into four domains: (a) individual characteristics, (b) individual health-related factors, (c) interpersonal/relationship to community resources, and (d) societal, policy, and governmental factors. Prior to the administration of the survey, participants were read the IRB-approved consent script and screened based on two questions: “How old are you?” and “Do you agree to participate in this study?” All of the survey participants were informed that the survey was voluntary, anonymous, would not affect services in any way, and that they could stop at any time during the survey process. On average, participants completed the survey within 45 min. Participants who completed the survey were offered a USD 20 gift card to local restaurants/services.

### 2.2. Measures

Dependent variables of depression. The dependent variables were measured as two dimensions of depression among homeless young adults: self-report depression scores and diagnosed depression. We examined self-reports of depression based on the PHQ-4 depression scale score [52]. This scale is composed of four items that ask respondents to rate how often they are bothered by “feeling nervous, anxious, or on edge”, “worrying”, “little interest or pleasure in doing things”, and “feeling down, depressed, or hopeless” over the last two weeks. Responses to the items were captured using a 4-point (0 to 3) scale, with higher scores indicating a greater number of days feeling bothered by the aforementioned problems. Responses for the four items were summed, with higher values indicating a greater severity of depression (normal [0–2], mild [3–5], moderate [6–8], and severe [9–12]) [52]. The PHQ-4 scale also has good internal consistency based on SDoH-HYA data, with an overall Cronbach’s alpha of 0.80.

The variable of diagnosed depression was measured by asking the participants whether they had ever been clinically diagnosed with depression by a doctor, nurse practitioner, or other healthcare professional. It was treated as a dichotomous variable (Yes = 1, No = 0). 

Variable of interest: ACEs. This study aims to examine the relationship between ACEs and depression among homeless young adults. We adopted the ACEs 10-item scale to measure childhood traumatic experiences [33] including physical abuse, verbal abuse, sexual abuse, physical neglect, and emotional neglect. In addition, the ACEs scale asks participants about family, such as a parent who’s an alcoholic, a mother who’s a victim of domestic violence, a family member in jail, a family member diagnosed with a mental illness, and the disappearance of a parent through divorce, death or abandonment. Each of the ACEs was dummy coded (Yes = 1, No = 0). Responses for the 10 items were summed, with higher values indicating a greater severity of childhood traumatic experiences. The ACEs scale has good internal consistency based on SDoH-HYA data, with an overall Cronbach’s alpha of 0.82.

Measures of covariates. For this study, based on the wider determinants of health framework [51], we controlled for covariates from demographics, interpersonal, and socioeconomic/living environment levels (see detailed measurement information in Table 1). 

At the individual characteristic level, we included participants’ demographic information: age, gender identity, sexual orientation, race/ethnicity, marital status, employment status, and the highest level of education completed. We also recorded how many children study participants have, and any children currently living with them.

At the interpersonal and community level, we examined whether participants keep in touch with their support system (friends/family), regularly attend religious services, use social media, have experienced domestic violence (defined as any violence from an intimate partner), have a criminal history, and if they consider themselves to be homeless. At the socioeconomic/living environment levels, we examined whether participants felt they have sufficient income, whether aging out of foster care caused them to be homeless, whether they have enough food to eat, have health insurance, can access transportation to go anywhere they want to, have Internet access, have a smartphone to use, feel the available support services are adequate, experienced discrimination as a homeless person, and whether they can get the help that they need.

### 2.3. Analytic Strategies

Descriptive statistics were conducted to illustrate the sample characteristics (see Table 1). To understand the extent to which ACEs and depression outcomes were related while controlling for the covariates at the individual, interpersonal, and socioeconomic/living environment levels, logistic regression was used for the diagnosed depression outcome whereas ordinary least squares (OLS) regression was used for the self-reported depression score outcomes. A listwise deletion approach was used to handle the missing data. Stata 15.0 was used to conduct the data analyses.

## 3. Results

### 3.1. Descriptive Statistics

As shown in Table 1, based on respondents’ reported depression symptoms over the past two weeks using the PHQ-4 scale, the average score was 5.01 (SD = 3.59) out of a total of 12. Specifically, about 29.27% of participants’ scores indicated a normal (0–2) depression status, 22.93% indicated mild (3–5) severity of depression, 30.73% indicated moderate (6–8) severity of depression, and 17.07% indicated severe (9–12) severity of depression. Meanwhile, 59.69% of respondents reported being diagnosed previously with depression. For this study, we were most interested in the relationship between depression outcomes and ACEs; the average score among our participants for the ACEs questionnaire was 5.22 (±2.97) out of a total of 10.

Overall, the average age of participants was 29.06 years old (±4.5 years) and the majority identified as male (66.67%). There were 16.58% of participants who identified as LGTBQIA+. The largest racial/ethnic group was White, non-Hispanic, followed by Native American and mixed race or other. The majority of participants had never married (63.37%) and have a high school diploma or GED (58.03%). In regard to employment status, only 9.05% have a full-time job while most are not employed but are looking for a job (42.71%). Nearly half have at least one child but only 8.87% are currently living with their child(ren).

We found that more than half of the participants try to keep in touch with their support system, such as friends and family. About one-third attend religious services regularly and a vast majority use social media of some kind. Domestic violence, defined as any violence from an intimate partner, was common for our participants (56.86%). Nearly half have a criminal history, such as history of incarceration, arrest, or accused of a crime. Only 64.39% consider themselves to be homeless.

Only 17.07% reported having enough sufficient income to support themselves and only 38.92% reported having enough food to eat. Of the reasons for becoming unstably housed or homeless, a few listed “aged out of foster care” as a reason (7.80%). Almost 9 in 10 participants have health insurance of some kind. Slightly more than half had the freedom to go anywhere that they want to and a majority had Internet access as well as a smartphone. Most participants felt that the support services that are available to them are adequate to meet their needs (63.68%). Slightly less than half of participants faced discrimination of some kind due to their perceived status as homeless. Many reported that they are able to access help if they need it (79.70%). 

### 3.2. Relationship between ACEs and Depression

Table 2 shows the two sets of regression results of testing the relationship between ACEs and depression outcomes, while controlling for all the covariates from the individual, interpersonal, and socioeconomic/living environment levels. Table 2, Column (a) shows the logistic regression results of the diagnosed depression outcome. Other things being equal, for every one unit increase in ACEs scores, the predicted odds of being diagnosed with depression increased by 11.5%, yet it was not statistically significant. Table 2, Column (b) shows the OLS regression results of PHQ-4 total depression scores. The results indicated that other things being equal, for every one unit increase in the ACEs score, the depression score increased by 0.445 (*p* < 0.001). 

For the individual-level demographic covariates, results showed that males had lower odds of being diagnosed with depression (OR = 0.174, *p* = 0.005) and lower self-reported depression scores than females. Homeless young adults who identified themselves as straight/heterosexual had significantly lower odds of being diagnosed with depression (OR = 0.043, *p* = 0.001) and lower self-reported depression scores (B = −1.214, *p* = 0.018) than LGBTQIA+ peers. As compared with White homeless young adults, Latinx (OR = 10.818, *p* = 0.035), Native American (OR = 11.204, *p* < 0.001), and multi-racial or other race group (OR = 5.771, *p* = 0.001) participants had significantly higher odds of being diagnosed with depression. However, only Native American (B = 0.15, *p* = 0.030) participants reported significantly higher depression scores than White participants. In addition, as compared with married homeless young adults or those in a relationship, those who were divorced or separated from partners (OR = 5.44, *p* = 0.03) or never married (OR = 4.185, *p* = 0.003) had significantly higher odds of being diagnosed with depression. Compared with full-time employed participants, those who were part-time employed (OR = 0.008, *p* < 0.001) or not employed and unable to work (OR = 0.035, *p* < 0.001) had significantly lower odds of being diagnosed with depression. Results also showed that homeless young adults that had children indicated significantly higher odds of being diagnosed with depression (OR = 3.877, *p* = 0.006), whereas those living with their children had significantly lower odds of being diagnosed with depression (OR = 0.064, *p* < 0.001). 

Regarding the interpersonal level covariates, results only showed that participants who had criminal history had significantly higher odds of being diagnosed with depression (OR = 2.693, *p* = 0.001). For socioeconomic and living environmental factors, homeless young adults who had enough food to eat (OR = 0.347, *p* < 0.001) and had Internet access (OR = 0.049, *p* < 0.001) showed significantly lower odds of being diagnosed with depression, compared with their counterparts. However, participants who reported that they had adequate support services had significantly higher odds of being diagnosed with depression (OR = 6.007, *p* < 0.001).

## 4. Discussion

This study adds further evidence to the research literature regarding the relationship between ACEs and depression outcomes among homeless young adults in the Phoenix metropolitan area, Arizona. We used two measures of depression outcomes as having been diagnosed with depression or as scores based on self-reported depression symptoms over the past two weeks. Overall, homeless young adult participants showed a higher level of risk of depression compared with the general young population [52,53]. For this survey, we found that about 60% of the participants had been diagnosed with depression, and nearly 70% of the participants’ depression scores indicate probable depression (i.e., showed mild severity of depression or worse). Before the COVID-19 pandemic, about 10%–20% of the general U.S. young adults had been diagnosed with depressive symptoms [54,55]. New data during and after the pandemic show that the prevalence of depression increased up to 17.2% among US young adults (18–25 years) [56]. The homeless population in our study is disproportionately affected by depression, which is consistent with the findings of other studies [57,58]. Results also showed that among homeless young adults, ACEs predicted greater severity of depression symptoms and higher odds of being diagnosed with depression. However, only the self-reported depression score outcome showed a statistically significant correlation with ACEs. A possible explanation of the non-significant relationship between ACEs and diagnosed depression is likely because compared with a self-reported depression score, obtaining a diagnosis of depression is a considerably higher standard. Many homeless young adults do not have access to or face other barriers to mental healthcare, and thus it is less possible for them to receive a diagnosis. In a study exploring the barriers to healthcare among people experiencing homelessness, interview responses identified barriers such as local mental healthcare providers not accepting new patients or public health insurance [59]. Therefore, the correlation between ACEs and diagnosed depression is likely underestimated.

Our survey results also showed a high proportion of respondents with health insurance (88%), which in line with previous studies’ results of 77% and 80% insured rates [60,61]. As reported by the United Health Foundation, the percentage of the general population in Arizona with health insurance coverage was approximately 89.3% [62]. Our study findings suggest that the uninsured rate within the young adult homeless population is consistent and close to the rate in the general population of Arizona, which could be explained by our study methodology of administering surveys in and around locations where community partners are providing social services. The relative ease of access to social services and resources is further corroborated by the result findings of 79.70% of respondents indicating that they “can get help if [they] need it”. This indicates that community partners and government agencies are achieving success in reducing some of the barriers to healthcare access among people experiencing homelessness.

Our study found that homeless young adults who identified as people of color had a higher risk of depression than their White counterparts. Native American homeless young adults had significantly higher odds of being diagnosed with depression and self-reported higher depression scores. This might be due to the majority of Native American participants receiving services from Solutions of Sobriety, which is an organization that provides recovery treatment services to Native American/Alaska Native tribal members who are seeking to recover from alcoholism and/or substance abuse. These participants have more opportunities to receive formal mental health diagnoses, including depression, when receiving services from Solutions of Sobriety. Nonetheless, other factors could explain the elevated rates of depression observed in this group. Native Americans/Alaska Natives report feelings of serious psychological distress greater than 2.5 times the general population [63]. Another study demonstrated a high prevalence of ACEs and a dose–response relationship between ACEs and negative mental health outcomes [48].

Our study also found that compared with LGBTQIA+ homeless young adults, those who identified themselves as straight/heterosexual had significantly lower odds of being diagnosed with depression by 93.7% and lower self-reported depression scores. This is consistent with previous studies which indicate that LGBTQIA+ homeless young adults are at higher risk for depression symptoms than their straight/heterosexual counterparts [64,65,66]. Study results also demonstrated that males had lower odds of being diagnosed with depression than females, which is consistent with various studies examining the general population [67]. 

We also found that homeless young adults who had full-time jobs had higher odds of depression than those with part-time jobs (OR = 0.008, *p* < 0.001) and those not employed and unable to work (OR = 0.035, *p* < 0.001). Those with full-time jobs usually complete assessments or screenings before they can be hired. Such assessments usually include questions about their background information, physical health, and mental health status including a depression screening [68]. Therefore, this might increase the diagnosis rate of depression among those with full-time jobs. In addition, those who work full-time in a workplace environment might face more stigma and therefore might increase their risk of depression. Full-time employment tends to come with more demanding responsibilities and stressors to maintain a full-time position, and, as previous studies have shown, there is an association between working longer hours and increased rates of depression [69,70]. Furthermore, those not employed but looking for a job did not have a significant difference compared to those with full-time employment. An explanation could be the increased stressors of attempting to secure employment while being discriminated against as homeless. This is juxtaposed with the situation of those not employed and unable to work; hence, people in this situation do not have to deal with the stressors of trying to find employment. This aligns with a study showing a significant relationship between depression and unemployment in emerging adults (ages 18–25), possibly due to delayed achievement of development goals related to the transition into adulthood and employment stability [71].

In regard to marital/relationship status, compared with respondents who are married or in a relationship, those who divorced or separated from partners and never married had significantly higher odds of being diagnosed with depression. This suggests that people in a supportive relationship have better mental health status versus those divorced or separated. The same holds true for those who are single since they have to manage the burdens of homelessness alone. It is notable that homeless young adults who had children reported significantly higher odds of being diagnosed with depression. However, those living with their children had significantly lower odds of being diagnosed with depression. Separating a parent from their children is a risk factor for depression [72]. We have also found a few other unique statistics with limited previous research that suggest the strong impact of close family relationships and spouses as protective factors against depression in homeless young adults, and inversely the increased risk of depression in these populations when faced with divorce or never married status (*p* = 0.020 and *p* = 0.003, respectively). This suggests that in the lens of homelessness, especially during young adulthood, isolation from loved ones or relationships may be playing a significant role in the elevated levels of depression in this population. This may be further true when considering the significantly elevated levels of depression in homeless young adults with a criminal history (*p* = 0.001), who may face similar isolation levels not just from personal relationships but on a societal scale, as they are discriminated against in job searches.

Moreover, the study findings revealed that those who had Internet access showed significantly lower odds of being diagnosed with depression. Along with the finding that the majority of respondents used social media of some kind, this supports the idea that better access to resources and support networks serve as protective factors against depression. However, it is important to note that there was no significant difference in risk among those who keep in touch with their support system (friends/family) and those who use social media.

We found the mean ACEs score to be 5.22 ± 2.97, which is consistent with Liu et al. who found an average of 4.05 and 4.41 for Canadian individuals experiencing homelessness amongst ages 18–24 and 25–44, respectively [47]. This number is unsurprisingly elevated when compared to the general United States population average of 1.56 [38]. The relationship between ACEs scores and homelessness is likely multifaceted and cyclic in nature. For example, an individual who has faced childhood abuse and dysfunction within their family may be more likely to experience homelessness; however, experiencing homelessness at a young age is also likely to increase ACEs scores as many of the factors in the ACEs questionnaire are more prominent in the unhoused community.

Further evaluating the impact of ACEs scores on depression, as mentioned prior, our results demonstrated a 0.445-point increase in PHQ-4 score for every one unit increase in ACEs score (*p* < 0.001). When further divided by demographic, this value was particularly staggering for those Native Americans with a significant 1.277-point increase in PHQ-4 score for every one unit increase in the ACEs score. Smith et al. also found individuals experiencing homelessness who identified as Indigenous and reported mental illness experienced significantly higher ACEs scores than those who did not [48]. While we consider those experiencing homelessness as a marginalized community, it is crucial to understand the intersectionality of identities within this group and the unique stressors each of those identities carries with it. Within the study, insufficient access to specific resources was also observed as a factor in determining the likelihood of depression in homeless young adults. In particular, we found that those with food insecurity (*p* < 0.001), those without Internet access (*p* < 0.001), and those without adequate support services (*p* < 0.001) were all at increased risk of having depression. These factors are of particular importance as unlike previous variables, these are controllable and with improved social programs could be potentially addressed. This may suggest that improving homeless young adult access to consistent nutritious meals would have not only a beneficial effect on overall physical health but on mental health and depression as well. Further study should focus on interventions that can effectively improve homeless young adults’ access to these resources.

This study has several limitations. First, this study used a convenient sample given the feasibility of reaching out to this population, and, therefore, the findings cannot make generalizations to the entire homeless young adult population. The study sample was limited to Arizona, and, therefore, caution is needed regarding generalizations to other geographic areas. Second, this study did not apply data imputation for missing data because of the non-probability sample. Third, self-reported recall information regarding an individual’s previous traumatized experiences or mental health status could be biased. Fourth, the study is prone to disadvantages embedded with the cross-sectional study design, in which data collection on depression outcome and independent variables were collected at the same time, and thus no causal inference should be drawn. Last, our participation pool was limited by the ability of participants to verbally communicate or read in English; future iterations of this research should include surveys in other regionally-prominent languages, such as Spanish.

Despite the limitations, this study has a number of strengths. This study used an SDoH perspective and included a comprehensive measure of factors from the individual, interpersonal, socioeconomic, and living environmental levels in the final regression models. This yields a holistic and better understanding of the risk and protective factors in the relationship between ACEs and depression outcomes. In addition, this study used two measures of depression: self-reported depression scores (subjective measure) and having been diagnosed with depression (objective measure). Using both subjective and objective measures of depression reduces social desirability biases of self-reported measures alone, and the results of this study are more robust.

Developing effective strategies to prevent and reduce homelessness has significant long-term implications for the public health, social and economic welfare, and equality of the United States. We propose that better social and medical programs on ACEs impact reduction be developed to empower people of homeless minority race/ethnicity groups, young parents, and LGBTQ+ groups. It is possible that regional factors played a significant role in why these specific groups were more/less affected in the homeless young adult population of Phoenix, Arizona, but it is also possible that specific cultural phenomena may be at play as well.

## 5. Conclusions

Our study elucidates the complex relationship between ACEs and depression among young adults experiencing homelessness, which contributes further evidence to the limited body of public health research involving homelessness in America. We found that self-reported and diagnosed depression and symptoms were disproportionally higher among the homeless population. ACEs were significantly associated with higher odds of diagnosed depression but not for self-reported measures. Within the Phoenix homeless population, members of race/ethnicity and sexual minority groups, unmarried people, and young parents were more vulnerable to depression and its risk factors. Future studies should examine the efficacy of interventions aimed at addressing the most prevalent correlates of depression from the perspective of SDoH, including lack of mental health services, lack of resources including food and Internet access, and lack of social support. Inputs from multiple stakeholders, including health professionals, social workers, and individuals who have or are currently experiencing homelessness, should be included in formulating the interventions.

## Figures and Tables

**Table 1:** Sample descriptive statistics.

(1)Variables	(2) Measures	(3) Mean/%	(4) *SD*
***Dependent Variables***			
PHQ-4 for depression	Total score (0–12)	5.01	3.59
Normal/minimal depression	Score 0–2 on PHQ-4	29.27%	
Mild depression	Score 3–5 on PHQ-4	22.93%	
Moderate depression	Score 6–8 on PHQ-4	30.73%	
Severe depression	Score 9–12 on PHQ-4	17.07%	
Depression diagnosis	Yes = 1; no = 0	59.69%	
***Variable of interest***			
Adverse childhood experiences (ACEs)	Total score (0–10)	5.22	2.97
***Covariate*** *s* **Demographics**			
Age	By year (18–34)	29.06	4.5
Gender identity	Male = 1; female = 0	66.67%	
Sexual orientation	Straight = 1; others = 0	83.42%	
**Race/Ethnicity**			
White	Yes = 1; no = 0	31.71%	
Black	Yes = 1; no = 0	13.17%	
Latinx	Yes = 1; no = 0	14.15%	
Native American	Yes = 1; no = 0	22.93%	
Mixed or other	Yes = 1; no = 0	18.05%	
**Marital Status**			
Married or in-relationship	Yes = 1; no = 0	18.32%	
Divorced or separated	Yes = 1; no = 0	18.32%	
Never married	Yes = 1; no = 0	63.37%	
**Employment Status**			
Fully employed	Yes = 1; no = 0	9.05%	
Part-time job	Yes = 1; no = 0	13.57%	
Not employed but looking for job	Yes = 1; no = 0	42.71%	
Not employed and unable to work	Yes = 1; no = 0	34.67%	
**Education**			
Less than high school	Yes = 1; no = 0	24.87%	
High school	Yes = 1; no = 0	58.03%	
Some college and higher	Yes = 1; no = 0	17.10%	
Have child/children	Yes = 1; no = 0	49.27%	
Living with child/children	Yes = 1; no = 0	8.78%	
**Interpersonal** Family and friends support	Yes = 1; no = 0	54.95%	
Attend religious services	Yes = 1; no = 0	29.06%	
Use social media	Yes = 1; no = 0	78.92%	
Domestic violence	Yes = 1; no = 0	56.86%	
Criminal history	Yes = 1; no = 0	48.98%	
Self-perception as homeless	Yes = 1; no = 0	64.39%	
**Socioeconomic/Living Environment** Sufficient income	Yes = 1; no = 0	17.07%	
Aged out of foster care	Yes = 1; no = 0	7.80%	
Enough food to eat	Yes = 1; no = 0	38.92%	
Have health insurance	Yes = 1; no = 0	88.00%	
Can go anywhere (transportation)	Yes = 1; no = 0	53.92%	
Had internet Access	Yes = 1; no = 0	64.71%	
Had smartphone	Yes = 1; no = 0	62.25%	
Adequate support services	Yes = 1; no = 0	63.68%	
Social discrimination	Yes = 1; no = 0	42.08%	
Can get help	Yes = 1; no = 0	79.70%	

**Table 2:** Regression results of ACEs and depression.

Variables	(a) Diagnosed Depression			(b) PHQ-4 Total Scores		
	OR	[95% CI]	*p*	Coef.	[95% CI]	*p*
***Variable of interest*** ACEs	1.115	[0.970, 1.281]	0.125	**0.433**	**[0.271, 0.595]**	**0.000**
***Covariates*** ***Demographics*** Age	0.916	[0.765, 1.097]	0.342	−0.025	[−0.193, 0.144]	0.753
Gender identity (Male = 1)	**0.174**	**[0.052, 0.589]**	**0.005**	1.014	[−0.359, 2.387]	0.133
Sexual orientation (Straight = 1)	**0.043**	**[0.007, 0.279]**	**0.001**	**−1.192**	**[−2.274, −0.110]**	**0.033**
**Race/Ethnicity (ref. = White)** Black	0.300	[0.041, 2.214]	0.238	0.998	−[1.852, 3.849]	0.460
Latinx	**10.818**	**[1.181, 99.071]**	**0.035**	0.950	[−0.037, 1.936]	0.058
Native American	**11.204**	**[3.352, 37.449]**	**0.000**	**1.277**	**[0.252, 2.302]**	**0.019**
Mixed or other	**5.771**	**[2.083, 15.988]**	**0.001**	0.646	[−0.888, 2.179	0.377
**Marital Status (ref. = married or in relationship)** Divorced or separated	**5.440**	**[1.305, 22.667]**	**0.020**	1.007	[−1.006, 3.020]	0.297
Never married	**4.185**	**[1.638, 10.696]**	**0.003**	0.063	[−0.808, 0.933]	0.878
**Employment status (ref. = full-time job)** Part-time job	**0.008**	**[0.001, 0.054]**	**0.000**	−0.244	[−3.785, 3.298]	0.883
Not employed but looking for job	0.246	[0.016, 3.668]	0.309	1.027	[−1.874, 3.928]	0.455
Not employed and unable to work	**0.035**	**[0.005, 0.228]**	**0.000**	1.529	[−1.286, 4.345]	0.260
**Education (ref. = some college or higher)** Less than high school	0.741	[0.303, 1.816]	0.513	−0.676	[−2.361, 1.010]	0.399
High school	0.594	[0.183, 1.922]	0.384	0.247	[−1.382, 1.877]	0.746
Have child/children	**3.877**	**[1.481, 10.149]**	**0.006**	−0.131	[−1.523, 1.260]	0.840
Living with child/children	**0.064**	**[0.019, 0.210]**	**0.000**	−0.499	[−3.114, 2.117]	0.685
***Interpersonal*** Family and friends support	2.823	[0.698, 11.412]	0.145	−1.061	[−2.254, 0.133]	0.077
Attend religious services	2.362	[0.463, 12.047]	0.301	0.591	[−1.914, 3.096]	0.617
Domestic violence	2.423	[0.444, 13.222]	0.307	0.357	[−0.443, 1.158]	0.350
Use social media	1.182	[0.241, 5.792]	0.836	−0.385	[−1.837, 1.068]	0.575
Criminal history	**2.693**	**[1.485, 4.884]**	**0.001**	−0.044	[−1.191, 1.103]	0.934
Self-perception as homeless	1.339	[0.412, 4.354]	0.628	1.057	[−0.005, 2.120]	0.051
***Socioeconomic/Living environment*** Sufficient income	0.747	[0.191, 2.922]	0.676	1.054	[−0.444, 2.552]	0.151
Aged out of foster care	2.565	[0.070, 93.908]	0.608	0.342	[−1.175, 1.860]	0.632
Enough food to eat	**0.347**	**[0.239, 0.506]**	**0.000**	−0.432	[−2.170, 1.306]	0.598
Have health insurance	5.998	[0.875, 41.142]	0.068	1.778	[−0.215, 3.770]	0.076
Can go anywhere (transportation)	1.223	[0.365, 4.098]	0.744	−0.149	[−1.299, 1.001]	0.782
Had Internet access	**0.049**	**[0.017, 0.141]**	**0.000**	0.650	[−0.484, 1.783]	0.236
Had smartphone	1.036	[0.418, 2.568]	0.940	−0.867	[−1.774, 0.039]	0.059
Adequate support services	**6.007**	**[2.521, 14.314]**	**0.000**	−0.605	[−1.467, 0.257]	0.152
Social discrimination	2.369	[0.761, 7.377]	0.137	0.733	[−0.819, 2.285]	0.324
Can get help	0.369	[0.024, 5.559]	0.471	−1.147	[−2.546, 0.253]	0.100
Constant	**57.870**	**[1.963, 1706.1]**	**0.019**	1.391	[−5.159, 7.941]	0.652

Notes: OR: odds ratio, CI: confidence interval, significant ORs (*p* < 0.05) were in bold.

## Data Availability

The authors elect not to share the data.
